# Méningite à streptocoque du groupe A chez le nouveau né: à propos d'un cas

**DOI:** 10.11604/pamj.2015.20.374.6141

**Published:** 2015-04-16

**Authors:** Hind El Youssi, Aziz Touaoussa, Hanouf Deham, Ghita Yahyaoui, Mustapha Mahmoud

**Affiliations:** 1 Laboratoire de Microbiologie CHU Hassan II, Fès, Maroc

**Keywords:** Méningite, nouveau né, ponction lombaire, streptocoque du groupe A, Meningitis, newborn, lumbar puncture, Group A streptococcus

## Abstract

Bien qu'une augmentation de l'incidence et de la sévérité des infections invasives au streptocoque du groupe A (SGA) durant ces dernières décades ait été constatée par plusieurs auteurs, les méningites dues à cette bactérie restent exceptionnelles chez le nouveau né et leur physiopathologie est encore mal connue. A travers ce travail nous allons rapporter le cas d'une méningite néonatale à SGA diagnostiquée au sein de notre formation, avec revue de la littérature s'intéressant à ces méningites.

## Introduction

Le streptocoque du groupe A est une cause rare de la méningite néonatale. Nous rapportons le cas d'un nouveau né atteint d'une méningite à streptocoque du groupe A diagnostiquée au laboratoire de microbiologie du CHU Hassan II de Fès.

## Patient et observation

Un nouveau né de sexe masculin issu d'une grossesse non suivie avec accouchement à domicile et bonne adaptation à la vie extra utérine, qui a présenté à j 22 de vie une fièvre avec refus de téter. L'examen initial trouve un nouveau né hypotonique, fontanelle antérieure bombante et température à 39°C. La ponction lombaire a ramené un liquide trouble avec la présence à l'examen direct de 750 GB/mm^3^ dont 90% de PNN. La coloration de Gram a révélé la présence de cocci gram positif en chainettes évoquant un streptocoque. L'examen biochimique a montré une gylcorachie à 0,25g/l (glycémie concomitante 1,01g/l) et des protides à 1.03 g/l. Après 24h la culture du LCR a révélé un streptocoque béta hémolytique identifié au groupe A par les techniques usuelles (test d'agglutination au latex, identification biochimique par galerie api 20 strep biomérieux et sur automate Phoenix) sensible à l'ensemble des antibiotiques testés selon les recommandations du CASFM. L'hémoculture réalisée le jour même est restée négative. Une antibiothérapie probabiliste à dose méningée à été démarrée associant une céphalosporine de 3^ème^ génération à un aminoside ayant été adaptée après résultat bactériologique par une amoxicilline. Après 48h du traitement le nouveau né s'est amélioré cliniquement avec apyrexie et reprise du tonus et de l'appétit. Une PL de contrôle a montrée une leucorachie à 125 GB/mm^3^ dont 65% de PNN sans germes à l'examen direct ni à la culture. Après 10 jours d'antibiothérapie intraveineuse le nouveau né est sorti en bon état clinique.

## Discussion

Avant la découverte des antibiotiques, le streptocoque du groupe A(SGA) était une cause majeure des septicémies néonatale et des infections puerpérales; les méningites représentaient 10 à 20% de ces infections avec un taux de mortalité atteignant 95%. Cependant depuis l'avènement de la thérapie antimicrobienne ces méningites sont devenues de plus en plus rares chez les enfants et les adultes [1]. Depuis les années 1980 une augmentation de l'incidence des infections invasives causées par le SGA a été notée [2]. Néanmoins, la méningite à SGA reste une entité rare qui peut affecter n'importe quelle tranche d'âge, des nouveaux nés aux adultes. Aux Etats Unis elle représente moins de 1% de toutes les méningites bactériennes de l'enfant [3] et environ 0.3% au pays bas [4]. Au brésil, le SGA était à l'origine de 0.9% de l'ensemble des méningites à germes identifiés de 1997 à 2007 [5]. A l'hôpital d'enfants de Casablanca parmi 751 méningites bactériennes à germes identifiés recensées entre 2003 et 2009 10 cas étaient dus à SGA soit environ 0.6% dont un nouveau-né à j5 de vie alors que les infections invasives à SGA ont atteint durant la même période un taux de 2.5% [6]. Dans la littérature anglo-saxonne uniquement 16 cas de méningite néonatale à SGA ont été rapportés depuis 1966 [7]. Parmi les 16 cas 2 patients avaient un âge inférieur à 7 jours. En 2004, dans une revue de la littérature s'intéressant aux infections invasives dues au SGA chez le nouveau né, 39 patients ont été décrits depuis 1966 [8]. La majorité des nouveaux nés, 24 (62%), avait une infection invasive à SGA à début précoce (< j5 de vie). La transmission verticale a été documentée dans 75% des cas; cependant le mode de transmission est resté inconnu dans les autres cas. La nature fulminante et les facteurs maternels concomitants indiquent que l'infection invasive à début précoce peut être la manifestation d'une diffusion hématogène ou d'un syndrome de médiation de toxine avec apparition in utéro [8]. L'apparition tardive de l'infection invasive à SGA (>j5 de vie) a été notée chez 15 (38%) nouveaux nés; le mode de transmission est inconnu dans la plupart des cas, uniquement quatre cas de transmission verticale ont été retrouvés. Ces résultats pourraient suggérer que la maladie d'apparition tardive est une manifestation relative à une petite charge bactérienne transmise verticalement ou à une acquisition post natale d'une infection localisée à SGA [8]. La période d'incubation de la plupart des infections streptococciques est très courte, habituellement de un à trois jours [9]. La transmission du SGA de personne à personne à l'intérieur d'une famille est bien connue. Le SGA peut se transmettre par un contact qui peut être direct ou indirect. La transmission par contact direct se fait par le transfert direct d'agents infectieux par un contact physique entre une personne colonisée ou infectée à un hôte réceptif. La transmission par contact indirect se fait par le transfert passif d'agents infectieux à un hôte réceptif par l'intermédiaire de matières inanimées ou d'objets contaminés. Les infections respiratoires à SGA se transmettent par l'exposition d'une personne réceptive (muqueuse nasale ou orale) aux gouttelettes respiratoires d'une personne infectée. Pour les plaies et les lésions cutanées infectées par le SGA, la transmission se fait par contact direct ou indirect entre l'écoulement (exsudat, pus ou tissu nécrotique) de la plaie ou de la lésion cutanée et la peau ou la muqueuse non intacte d'une personne réceptive [9].

Cependant, la physiopathologie de l'infection méningée primitive à SGA reste peu claire. Une partie de ces méningites peut être expliquée par la diffusion (locale ou hématogène) d'une infection localisée. Néanmoins, environ 30% des méningites à SGA décrites dans la littérature restent inexpliquées [10]. Parmi 53 cas de méningites à SGA rapportés dans le continent américain, la porte d'entrée était ORL dans 45% des cas, cutanée dans 11% des cas dont deux cas de varicelle et dans deux cas la porte d'entrée a été relative à un implant cochléaire [6]. Comme pour toutes les infections invasive à SGA, le développement d'une méningite peut être lié à des facteurs de l'hôte et/ou à des facteurs de virulence du pathogène. Plusieurs facteurs reliés à l'hôte ont été associés à un risque accru d'infection invasive à SGA: maladie cardiaque, diabète, cancer, infection par le VIH, varicelle, maladie pulmonaire chronique, lésion cutanée, chirurgie de la sphère ORL, etc. [9]. En effet l'état immunitaire au moment d'une infection influence le risque d'infection invasive à SGA (ex: présence ou absence d'anticorps protecteurs contre les différents types de protéine M en cause, présence ou absence d'anticorps contre certaines exotoxines, immunodéficience) [9]. De plus des facteurs génétiques confèrent une protection contre des agents infectieux ou au contraire entraînent une prédisposition à des agents, car des personnes infectées par une même souche de SGA peuvent présenter des manifestations cliniques de gravité différente [9]. Les SGA sont pourvus de facteurs de virulence leur permettant d'envahir les tissus de l'organisme, d'échapper au système immunitaire ou de le perturber par une activation non spécifique (exotoxines pyrogéniques) [11]. Récemment, le rôle d'un complexe trimoléculaire combinant le plasminogène au fibrinogène et à la streptokinase a été associé à la dissémination tissulaire du SGA [11]. De nombreux facteurs de virulence vont jouer un rôle protecteur contre le système immunitaire: protection contre l'opsonisation et la phagocytose grâce à la capsule d'acide hyaluronique, la protéine M et le facteur Mac-1/2, inhibition du chimiotactisme des polynucléaires neutrophiles grâce aux protéases ScpA (C5a-peptidase) et ScpC (protéase inactivant l'IL-8), résistance aux peptides antibiotiques (protéase SpeB et inhibiteur du complément SIC), lyse des phagocytes (streptolysines S et O), échappement aux pièges à ADN des polynucléaires neutrophiles (DNAses) [11]. Onze superantigènes ont actuellement été décrits chez S. pyogènes [11]. L'existence de clones de SGA plus virulents que d'autres fait encore débat. Si certaines études ne mettent pas en évidence de différences significatives entres souches responsables d'infections focales bénignes et souches d'infections invasives [11], d'autres montrent la prédominance de certains facteurs de virulence (toxine érythrogène SpeA, inhibiteur du complément SIC) ou types de protéine M (emm1/M1, emm3/M3) chez les souches de SGA responsables d'infections invasives ou bien les associent à un pronostic plus sévère [11]. Notamment, un clone de sérotype M1 de diffusion mondiale a été associé à l'augmentation des infections invasives à SGA [11]. D'autres clones invasifs, associés à des gènes de virulence particuliers ont été décrits, comme les gènes du locus sil chez des souches M14 isolées en Israël [11].

Enfin, des facteurs reliés à l'environnement peuvent favoriser la transmission du SGA. Le fait d'être un contact étroit d'un cas d'infection invasive à SGA augmente le risque de contracter cette infection. En établissement de soins, la difficulté à appliquer les mesures préventives requises peut contribuer à la transmission du SGA. La proximité, particulièrement en milieu fermé, peut augmenter le risque de transmission du SGA [9]. Dans notre observation il n'y avait pas de maladie sous jacente ni aucun autre facteur prédisposant à la méningite au SGA. Les antibiotiques utilisés dans le traitement des infections à SGA appartiennent principalement à la famille des béta lactamines et à celle des macrolides, lincosamides et synergistines (MLS) [11]. Aucune souche de SGA résistante aux béta lactamines n'a jamais été décrite jusqu'à ce jour. Cependant, la résistance des souches à la famille des MLS pose problème, et le pourcentage de souches résistantes et les mécanismes de résistance impliqués varient considérablement d'un pays à l'autre et au cours du temps, en fonction de la consommation d'antibiotiques, ce qui impose une surveillance régulière de la sensibilité des souches responsables d'angines ou de pathologies invasives afin de guider les recommandations d'antibiothérapie de 1^ère^ intention. Par ailleurs la pénicilline reste l'ATB de choix pour la méningite à SGA. Les C3G constituent une bonne alternative en cas de complications suppurative en raison de la meilleure capacité de diffusion. Dans la série anglo-saxonne de 16 nouveaux nés la pénicilline a été utilisée dans 77% [7]. Les autres antibiotiques utilisés inclus aminosides, céphalosporine de deuxième et troisième génération, et la vancomycine.

L´évolution clinique rapportée par la littérature est très diverse ; concernant les 16 cas rapportés dans la littérature anglo-saxonne l'évolution clinique a été associée à des complications majeures y compris des convulsions chez 8 patients (60%), une coagulopathie intravasculaire disséminée (CIVD), une insuffisance cardio-respiratoire, une hépatite, un syndrome de Waterhouse-Frederichsen et trois patients ont développé un abcès cérébral. Huit patients (50%) sont décédés [7]. C´est plus que le taux de mortalité des nouveau-nés atteints de méningites causés par autres agents pathogènes, et le taux de mortalité des enfants atteints de méningite à SGA au-delà de la période néonatale [12]. Le seul nouveau né atteint d'une méningite à SGA, rapporté dans la série casablancaise de 10 cas, est décédé dans un tableau de choc septique [6]. Dans notre cas le patient a été initialement traité par C3G associé à un aminoside relayés par une amoxicilline. L'évolution était bonne sans séquelles neurologiques relatives à la méningite.

## Conclusion

La méningite à SGA est une entité rare chez le nouveau né, elle est généralement de bon pronostic mais une issue fatale peut survenir même chez des enfants sans facteur de risque d'infection grave identifié. La vaccination contre le SGA actuellement en cours de développement, pourrait à l'avenir être utile pour prévenir les infections invasives secondaires au SGA en particulier la forme méningée et également pour la prévention des cas secondaires. Ce type de vaccin nécessite de connaitre les types de souches circulant afin de juger de son efficacité et de détecter un éventuel changement épidémiologique en cour de vaccination [12].

## References

[1] Murphy DJ (1983). Group A Streptococcal meningitis. Pediatrics..

[2] Roger S, Commons R, Danchin MH, Selvaraj G, Kelpie L, Curtis N (2007). Strain prevalence, rather than innate virulence potential, is the major factor responsible for an increase in serious group A streptococcus infections. J Infect Dis..

[3] Mariana V, Arnoni A, Eitan N, Berezin A, Marco AP (2007). Streptococcus pyogenes meningitis in children: report of two cases and literature review. Braz J Infect Dis..

[4] Van de Beek D, Gans J, Spanjaard L (2002). Group a streptococcal meningitis in adults: report of 41 cases and a review of the literature. Clin Infect Dis..

[5] Santos MS, Ribeiro GS, Oliveira TQ, Santos RN (2009). Streptococcal meningitis in Salvador Brazil: report of 11 years of population-based surveillance. Int J Infect Dis..

[6] Jouhadi Z, Sadiki H, Lehlimi M, Honsali Z, Najib J (2012). Meningites à streptocoque du groupe A. Médecine et maladies infectieuses..

[7] Lardhi AA (2008). Neonatal group A streptococcal meningitis: a case report and review of the literature. Cases Journal..

[8] Miyairi I, Berlingieri D, Protic J, Belko J (2004). Neonatal invasive group A Streptococcal disease: case report and review of the literature. Pediatr Infect Dis J..

[9] Frignon M, Lagarde G, Perron L (2012). Les infections invasives à streptocoques du groupe A. Guide d'intervention. Mise à jour.

[10] Hentgen V, Levy C, Bingen E, Cohen R (2008). Méningite à streptocoque A : caractéristiques d'une méningite rare chez l'enfant. Arch Pediatr..

[11] Bidet P, Plainvert C, Doit C, Kurkdjian PM, Bonacorsi S (2010). Infections à Streptococcus pyogenes ou streptocoque du groupe A chez l'enfant : données du Centre national de référence (CNR). Archives de pédiatrie..

[12] Shetty AK, Frankel LR, Maldonado Y, Falco DA, Lewis DB (2001). Group A Streptococcal meningitis: Report of a case and review of literature since 1976. Pediatr Emerg Care..

